# Synchronous colorectal adenocarcinoma and gastrointestinal stromal tumor in Meckel's diverticulum; an unusual association

**DOI:** 10.1186/1477-7819-7-33

**Published:** 2009-03-23

**Authors:** Christopher Kosmidis, Christopher Efthimiadis, Sofia Levva, George Anthimidis, Sofia Baka, Marios Grigoriou, Ioanna Tzeveleki, Maria Masmanidou, Thomas Zaramboukas, Georgios Basdanis

**Affiliations:** 1 Department of Surgery, Interbalkan European Medical Center, Thessaloniki, Greece; 2 Department of Oncology, Interbalkan European Medical Center, Thessaloniki, Greece; 3 1st Propedeutic Surgical Clinic, Aristotle University of Thessaloniki, AHEPA Hospital, Thessaloniki, Greece; 4 1st Propedeutic Clinic of Internal Medicine, Aristotle University of Thessaloniki, AHEPA Hospital, Thessaloniki, Greece; 5 Pathology Department, Aristotle University of Thessaloniki, Thessaloniki, Greece

## Abstract

**Background:**

Coexistence of gastrointestinal stromal tumor with synchronous or metachronous colorectal cancer represents a phenomenon with increasing number of relative reports in the last 5 years. Synchronous occurence of GISTs with other gastrointestinal tumors of different histogenesis presents a special interest. We herein report a case of GIST in Meckel's diverticulum synchronous with colorectal adenocarcinoma.

**Case presentation:**

A 69 year old man, presented with abdominal distension and anal bleeding on defecation. Colonoscopy revealed colorectal cancer and a low anterior resection was performed, during which a tumor in Meckel's diverticulum was discovered. Histologic examination revealed GIST in Meckel's diverticulum and a rectosigmoid adenocarcinoma.

**Conclusion:**

Whenever GIST is encountered, the surgeon should be alert to recognize a possible coexistent tumor with different histological origin. Correct diagnosis of synchronous tumors of different origin is the cornerstone of treatment.

## Background

Meckel's diverticulum is surgically removed only when a complication arises or a neoplasia develops. Neoplastic transformation has been reported, but gastrointestinal stromal tumors (GISTs) are exceptional in this location [1,2]. The management of GISTs has dramatically evolved over the last ten years. However, their coexistence with other gastrointestinal tumors of different histogenesis presents a special clinical problem. We herein report a case of GIST in Meckel's diverticulum synchronous with colorectal adenocarcinoma.

## Case presentation

A 69 year-old-man presented with abdominal distension and anal bleeding on defecation. For the last six months the patient was complaining of flatulence, periumbilical pain and sensation of incomplete evacuation. Apart from tenderness on deep palpation, blood on PR examination and increased bowel sounds, all other systems were found normal on clinical examination.

**Figure 1:**
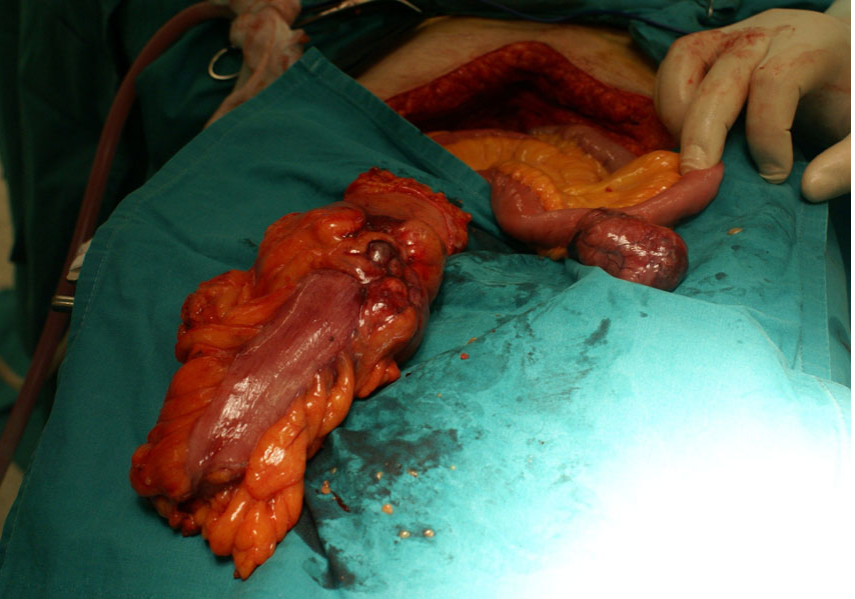
**The colorectal adenocarcinoma having been excised and the coexisted GIST in Meckel's diverticulum**.

**Figure 2:**
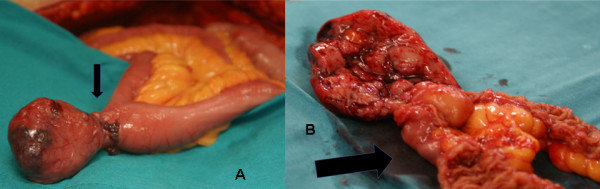
**A: The specimen of the GIST in Meckel's diverticulum**. The arrow shows the diverticulum. B: Transverse section of the specimen of the GIST in Meckel's diverticulum and the proximal part of the ileum. The arrow shows the diverticulum.

Colonoscopy revealed intraluminal stenosis of the colorectal junction and biopsy specimens were obtained. Biopsy confirmed a well differentiated mucinous adenocarcinoma of the rectosigmoid colon. There was no evidence of metastasis, based on abdominal computed tomography (CT), chest X-ray and endorectal ultrasound (US) 3D. Carcinoembryonic antigen (CEA), carbohydrate antigen (CA) 19-9, and cancer antigen (CA) 50 were normal. Low anterior resection for colorectal carcinoma (CRC) with an end to end primary anastomosis was performed, while on exploration a mass in Meckel's diverticulum, 80 cm proximal to the ileocecal valve, was encountered (Figure 1). The mass was 7.5 cm in maximal diameter (Figure 2A, B), while the size of the Meckel's diverticulum was 3 cm. Thus, this case is classified as an "intermediate risk" GIST according to the risk assessment of aggressive behavior in GISTs proposed by Fletcher et al. (size: 7.5 cm, mitotic count: 0-1/50 HPF) [3]. No evidence of liver metastasis or intra-abdominal metastatic spread was found. The patient underwent resection of the tumor together with partial resection of the small bowel in order to avoid rupture and intra-abdominal spillage. Tumor was excised, together with 3 cm of ileum on either side, and a lateral to lateral anastomosis was performed with the use of staplers.

**Figure 3:**
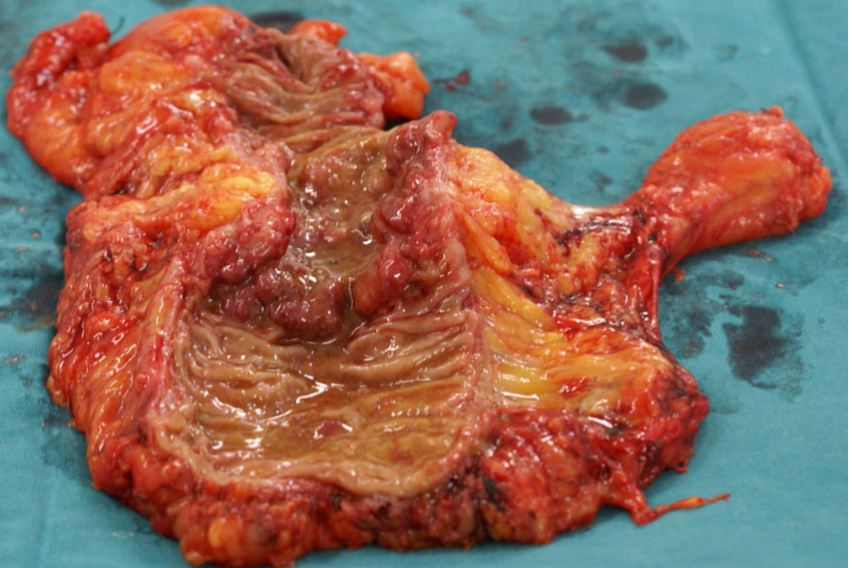
**The specimen of the colorectal adenocarcinoma**.

**Figure 4:**
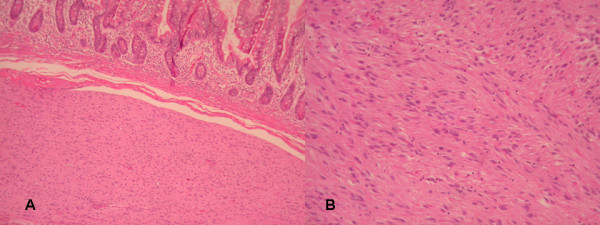
**A) Invasion of submucosa of small intestine from GIST (HE×200)**. B) Histologically, the GIST was composed of sheets of spindle cell with moderate to slight interstitial collagen (HE×200).

**Figure 5:**
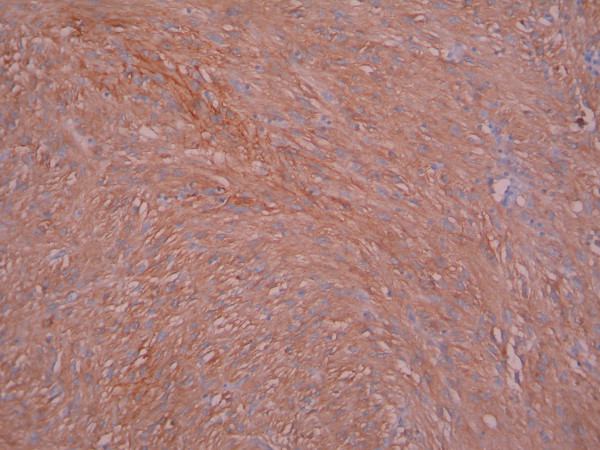
**Immunohistochemically, spindle cells showed cytoplasmic staining for CD117(c-kit) (×200)**.

The lesion in the colorectal junction was a stage C2 (Astler-Coller) tumour, 8 × 6 cm in size (Figure 3), located in the rectosigmoid colon. Microscopic examination showed a well-differentiated adenocarcinoma, penetrating the bowel wall, without nerve and vascular invasion. However, one of the 23 resected lymph nodes was positive for metastasis. According to the TNM (tumor, lymph nodes, metastasis) classification, the disease was stage IIIb. Histopathological examination of the resected Meckel's diverticulum tumor revealed a stromal cell neoplasm with a few necrotic and hemorrhagic areas and a low index of mitotic count (0–1 mitoses/50 HPF) (Figure 4A, B). Immunohistochemical analysis revealed expression of C-kit (strongly positive), moderately positive stain for SMA, while markers for focal S-100 and CD 34 were negative (Figure 5).

Patient's postoperative course was uneventful, and he was commenced on adjuvant chemotherapy with FOLFOX regimen (oxaliplatin i.v., 85 mg/m^2^(on day 1), leukovorin i.v., 200 mg/m^2 ^(on days 1,2), 5 FU (fluorouracil) i.v., bolus 400 mg/m^2 ^(on days 1,2), 5 FU i.v., 22 hours-infusion, 600 mg/m^2 ^(on days 1,2), every 2 weeks for 12 cycles.

One year later he has no signs of local recurrence or metastasis.

## Discussion

The term GIST was introduced by Mazur and Clark in 1983 in order to define a heterogeneous group of neoplasms of spindle and epithelioid cells arising from the stroma with no definite cell line of origin and varying patterns of differentiation [4,5]. GISTs are considered to be mesenchymal neoplasms, encompassing a majority of tumors previously considered gastrointestinal smooth muscle tumors, which, with the implementation of immunohistochemical stains and electron microscopy in recent years, have been recognized as a distinct pathological entity [3]. Their nomenclature, histogenesis, criteria for diagnosis, prognostic manifestations, and classification have raised much debate and controversy [6,7]. Though the most common mesenchymal tumors in the gastrointestinal tract wall, arising from Cajal's cells, they are rare neoplasms accounting for less than 1% of all gastrointestinal tumors [3].

The most common sites are stomach (60%) and small intestine (30%). The average presentation is in the sixth decade [8]. Most GISTs (>90%) express CD117, the c-*kit *proto-oncogene protein that is a transmembrane receptor for the stem cell growth factor, and 70% to 80% express CD34, the human progenitor cell antigen; less often, these tumors stain positive for actin and desmin [9].

The three most important factors in determining malignancy are mitotic rate, tumor size and site [8,10-12]. Mitotic counts higher than 2 per 50 high-power fields imply an increased risk for local recurrence in small bowel GIST [10]. It is generally accepted that the criteria for predicting biological behavior may differ significantly with location. For example gastric GISTs are less aggressive than those located in small bowel. In the small intestine there is an overall 39% tumor related mortality, twice that for gastric GISTs [8,13].

Smaller GISTs are often incidental findings during surgery, radiologic studies, or endoscopy. Coexisting stromal tumors are usually very small and are detected incidentally during gastrointestinal surgery for carcinomas [14]. In our case the patient presented with anal bleeding, which was attributed to the colorectal carcinoma. Preoperative evaluation did not reveal the unique coexistence of tumor in Meckel's diverticulum along with the rectosigmoid cancer, even though the tumor was large, 7.5 cm in maximal diameter.

The patient underwent an en-block resection of the tumor to avoid rupture and intra-abdominal spillage, as it is recommended [10]. He was commenced on adjuvant chemotherapy with oxaliplatin, leukovorin, and fluorouracil. Imatinib mesylate was not used since treatment of localized GIST is complete surgical excision, without dissection of lymph nodes [11,12] and adjuvant treatment with imatinib is still under consideration. Imatinib is the standard treatment if complete surgery is not feasible [11]. As the localized GIST was excised intact, there was no rationale to submit the patient to a treatment that should be continued indefinitely and the long-term success of which is limited by development of imatinib resistance via secondary mutations or clonal selection [11].

Despite the considerable development in the management of GISTs the last 10 years and the immense progress recently made in understanding the molecular biology of GISTs, little is yet known about their rare synchronous occurrence with tumors of different histogenesis. GISTs have been reported to occur synchronously with colon adenocarcinoma, gastric cancer, lymphoma and carcinoid [13,15-18]. The coexistence of GIST with either synchronous or metachronous colorectal cancer represents a rare phenomenon with increasing number of relative reports in the last 5 years.

Although the synchronous occurrence of GIST and other abdominal malignancy seems to be just a coincidence, combined genetic deregulation or influenced neighboring tissues by the same carcinogen could be causative factors [17,19]. There are some data regarding the co-occurrence, the association and the potential common origin (genetic pathways of tumorigenesis), between GIST and other tumors [18-20]. The limited number of these cases cannot confirm the existence of a common factor in tumorigenesis of these histopathologically completely different tumors and further studies are needed to clarify the possible association.

Most of the published cases describe gastric stromal tumors synchronous with another gastric malignancy. Our case describes a concomitant GIST in Meckel's diverticulum with a colorectal adenocarcinoma. As far as we could elicit from the literature, this is the first report of such tumor coexistence with regard to location.

## Conclusion

The role of thorough abdominal exploration despite advanced preoperative imaging techniques cannot be overemphasized. In case of colorectal cancer, the doctor should bear in mind the possibility of coexistent tumor with different histological origin elsewhere in the GI tract.

## Consent

Written informed consent was obtained from the patient for publication of this Case report and any accompanying images. A copy of the written consent is available for review by the Editor-in-Chief of this journal.

## Competing interests

The authors declare that they have no competing interests.

## Authors' contributions

KC and EC and BG performed the operation and together with AG and GM contributed to the conception and design of the manuscript. LS, BS, MM, and TI analyzed and interpreted the patient data regarding the disease. AG and LS were major contributors in writing the manuscript. ZT carried out the histology and immunohistochemistry examination. All authors read and approved the final manuscript.
